# A DNA Prime and MVA Boost Strategy Provides a Robust Immunity against Infectious Bronchitis Virus in Chickens

**DOI:** 10.3390/vaccines11020302

**Published:** 2023-01-30

**Authors:** Shaswath S. Chandrasekar, Brock A. Kingstad-Bakke, Chia-Wei Wu, Yashdeep Phanse, Jorge E. Osorio, Adel M. Talaat

**Affiliations:** 1 Department of Pathobiological Sciences, School of Veterinary Medicine, University of Wisconsin, Madison, WI 53706, USA; 2 Pan Genome Systems, Madison, WI 53719, USA; 3 Colombia Wisconsin One Health Consortium, Universidad Nacional Medellín, Calle 75#79a 51, Colombia

**Keywords:** IBV, nanovaccine, intranasal vaccine, heterologous vaccine

## Abstract

Infectious bronchitis (IB) is an acute respiratory disease of chickens caused by the avian coronavirus Infectious Bronchitis Virus (IBV). Modified Live Virus (MLV) vaccines used commercially can revert to virulence in the field, recombine with circulating serotypes, and cause tissue damage in vaccinated birds. Previously, we showed that a mucosal adjuvant system, QuilA-loaded Chitosan (QAC) nanoparticles encapsulating plasmid vaccine encoding for IBV nucleocapsid (N), is protective against IBV. Herein, we report a heterologous vaccination strategy against IBV, where QAC-encapsulated plasmid immunization is followed by Modified Vaccinia Ankara (MVA) immunization, both expressing the same IBV-N antigen. This strategy led to the initiation of robust T-cell responses. Birds immunized with the heterologous vaccine strategy had reduced clinical severity and >two-fold reduction in viral burden in lachrymal fluid and tracheal swabs post-challenge compared to priming and boosting with the MVA-vectored vaccine alone. The outcomes of this study indicate that the heterologous vaccine platform is more immunogenic and protective than a homologous MVA prime/boost vaccination strategy.

## 1. Introduction

Coronaviruses (CoVs) are enveloped, large viruses with a positive-sense, single-strand RNA genome ranging from 27–31 Kb in length. They are broadly classified into four genera: *Alphacoronavirus*, *Betacoronavirus*, *Gammacoronavirus*, and *Deltacoronavirus* [1]. CoVs can infect a wide range of hosts, including humans, poultry, mice, pigs, cats, camels, bats, etc. CoV infections usually cause acute diseases, primarily in the respiratory and gastrointestinal tracts [1]. Infectious Bronchitis Virus (IBV) is a *Gammacoronavirus* and in chickens, it can cause an acute respiratory disease called infectious bronchitis (IB) [1]. The IBV genome encodes five major structural proteins: spike glycoprotein (S), envelope (E), membrane (M), and nucleocapsid (N) [2]. In a typical infectious bronchitis infection, chickens develop respiratory signs, including sneezing, tracheal rales, nasal discharge, and labored breathing [3]. Mortality associated with infectious bronchitis is low; however, concomitant secondary bacterial infections can increase mortality [4]. Infectious bronchitis has a significant economic impact on the commercial US poultry industry, valued at over USD 30 billion per year in the US [5]. Infectious bronchitis infections in broilers can lead to reduced weight gain, and low feed conversion and infections in layers can lead to a drop in egg production and quality [6,7]. Typically, losses of around USD 450,000 per week can be expected due to IB outbreaks in facilities producing about 1 million broilers per week, which is unsustainable in the poultry industry, as it is characterized by low-profit margins [8]. IBV control currently revolves around extensive vaccination and acceptable flock management practices, such as optimal stocking densities, house temperature, water and air quality, etc., to prevent increased mortality due to secondary bacterial infections. Modified live virus (MLV) and inactivated vaccines are the leading vaccine types used against IB. Although effective, MLVs have an inferior safety profile and can cause tissue damage, especially in day old chicks [9]. MLVs have a propensity to persist, revert to virulence in the field, and readily recombine with other circulating serotypes, leading to the emergence of novel serotypes [10,11,12]. Moreover, current vaccines do not cross-protect against multiple circulating serotypes because of variations in the S protein [13,14,15]. Unfortunately, safer inactivated vaccines are poorly immunogenic, underscoring the need to develop an effective and safe vaccine for IBV control [8].

Experimental plasmid DNA vaccines have been developed against multiple poultry pathogens, and most recently, conditional approval for a DNA vaccine against H5 avian influenza was given [16]. Varying protection levels are observed with experimental plasmid DNA vaccines expressing IBV S1, N, and M genes delivered via the intramuscular, intranasal, and in ovo routes [17,18,19,20,21,22,23,24,25]. DNA vaccines offer several advantages over traditional vaccine approaches; they are safe, thermostable, comparatively inexpensive, and can be rapidly developed in the face of a novel serotype field outbreak [26]. A significant problem with DNA vaccines is their low immunogenicity owing to in vivo degradation, which leads to reduced cellular uptake and bioavailability. Vaccine hostile surfaces, such as the nasal mucosa, can degrade the DNA vaccine before target immune cell uptake [27,28]. Nanoparticle adjuvant systems such as QAC can protect DNA against degradation and boost the immune responses observed with DNA vaccines, as described by our group previously for the intranasal delivery of DNA immunogens [29,30].

Similarly, viral vector vaccines against IBV based on Newcastle disease virus and avian metapneumovirus backbones have been developed [31,32]. However, none of them have been licensed for use due to limited efficacy and regulatory concerns. Although the concept of a heterologous vaccine for the poultry industry refers to vaccination with a different serotype than the challenge virus, or priming and boosting with different serotypes with the goal of producing a broadly cross-protective response [33,34], for the purpose of this paper, a heterologous vaccination strategy refers to the concept of using a different vaccine platform for boosting from the vaccine that was used for priming. In this study, we particularly evaluated DNA priming followed by viral vector boosting in comparison to viral vector homologous priming and boosting. The heterologous prime–boost vaccination strategy has been evaluated against viral pathogens such as HIV-1, HPV, HCV, and influenza virus [33,34,35,36,37]. The efficacy of heterologous vaccine strategies has been shown with different routes and viral vectors for boosting, such as vaccinia virus (e.g., Modified Vaccinia Ankara—MVA), adenovirus, and VSV (Vesicular Stomatitis Virus) [38]. Multiple human clinical trials have indicated that the most effective prime–boost strategies involve a DNA vaccine prime followed by viral vector boosting, which leads to more robust immune responses, especially T-cell responses [38]. Heterologous vaccination, compared to homologous immunization, can lead to a 4- to 10-fold increase in T-cell responses [38]. In a Phase-I clinical trial, heterologous DNA priming followed by vaccinia viral vector boosting against HIV-1 induced longer-lasting and polyfunctional T-cells compared to the homologous vaccinia viral vector vaccine regimen [39]. Although the heterologous vaccine platform has been characterized and extensively evaluated for human viral pathogens, not much work has been done in the context of viral poultry pathogens.

We have previously shown that a two-dose QAC-encapsulated plasmid DNA (pQAC-N) vaccination encoding the N protein was protective against an IBV challenge to levels seen with MLV vaccination [30]. We hypothesized that a heterologous vaccine strategy with a pQAC-N prime followed by an MVA viral vector boost expressing the N protein (MVA-N) would also protect immunized chicks against IBV challenge. The prime/boost of the experimental vaccines were delivered via the intranasal route, and is hereafter referred to as either the heterologous vaccine strategy or pQAC/MVA-N. Our results indicate that the pQAC/MVA-N vaccine elicits robust, IBV-specific CD8+ and TCRγδ+ T-cell responses which protect vaccinated birds against IBV challenge. Levels of protection in vaccinated birds were higher when compared to the homologous 2X MVA-N vaccine. Our data demonstrate that intra-nasal immunization with pQAC/MVA-N protected vaccinated birds, with a significant reduction in clinical signs and viral load in trachea and lachrymal fluid to levels on par with commercial MLV-vaccinated birds. Furthermore, addition of another adjuvant MPLA (Synthetic Monophosphoryl Lipid A) did not significantly improve the level of protection observed with pQAC/MVA-N.

## 2. Materials and Methods

### 2.1. Ethics Statement

All the animals used in this study were cared for in accordance with established guidelines, and the experimental protocols were approved by the Institutional Animal Care and Use Committee (IACUC) of the University of Wisconsin at Madison.

### 2.2. Cells and Viruses

Chicken Embryonic Fibroblasts (CEF) were prepared from 9-day old embryos from specific pathogen-free (SPF) white leghorn eggs (Charles River Laboratories, Inc., Wilmington, MA, USA), as described previously [40], and were used to confirm expression of the IBV Ark N6xHis protein from vaccine constructs. The cells were cultured in DMEM (Dulbecco’s Modified Eagle Medium) at 37 °C and 5% CO_2_ atmosphere in plastic flasks with ventilated caps. The virulent IBV Arkansas DPI strain (a kind gift from Dr. Ladman and Dr. Gelb) was propagated in 9-day old SPF embryonated chicken eggs (ECEs) and allantoic fluid was harvested four days after infection. The stock virus titer was determined and expressed as 50% of the embryo infectious dose (EID_50_) [41]. The IBV S1 gene sequence of the Ark DPI challenge isolate is AF006624.

### 2.3. Preparation of IBV Vaccine Constructs

pCAG-N, encoding an IBV N protein matching the challenge strain, was loaded into QAC nanoparticles as described previously [30]. The MVA expressing N was generated as described for in CEF cells [42]. The cell and supernatant fractions were boiled in Laemmli sample buffer (BioRad, Hercules, CA, USA) and resolved on a 4–20% SDS-PAGE gel by electrophoresis using a Mini-PROTEAN 3 system (BIO-RAD, Hercules, CA, USA). Polyacrylamide gels were electroblotted onto nitrocellulose membranes using a Turboblot^®^ system. Membranes were blocked in 5% (*w*/*v*) skim milk and probed with polyclonal anti-6xHis HRP antibody (ThermoFisher Scientific, Waltham, MA, USA, MA1-21315-HRP). Membranes were developed using a solid phase 3, 30, 5, and 50 -tetramethylbenzidine (TMB) substrate system.

### 2.4. Vaccine Efficacy Study

The protective efficacy of pQAC/MVA-N construct was evaluated in 1-day old white leghorn SPF chicks (Charles River Laboratories). A total of 36 chicks were divided into 4 groups and used for the efficacy study. The first 2 groups (N = 8 each) were inoculated with PBS (negative control) or commercial Arkansas MLV (Mildvac-Ark^®^, Merck Animal Health, Madison, NJ, USA, positive control) via direct intranasal instillations (dose according to manufacturer’s instructions). The other groups (N = 10 each) were either vaccinated with MVA-N (10^8^ pfu/bird) at day 1 followed by a booster dose at day 14 via the intranasal (IN) route, or pQAC-N (100 µg/bird) at day 1 followed by a booster MVA-N (10^8^ pfu/bird) dose at day 14 via the intranasal (IN) route. Birds were challenged with a dose of 10^6.5^ EID_50_/bird of a virulent IBV Arkansas DPI strain via direct intranasal instillations at day 21 of age. The challenge dose was determined in an independent infection experiment wherein the challenge dose resulted in discernable clinical signs as early as 3 dpc and peak viral load replication was observed at 6 dpc. At 10 and 20 days post-prime vaccination (dpv) and 3 days post-challenge (dpc), serum and lachrymal fluid samples were harvested for ELISA, and this was repeated at 6 dpc for viral load estimation (see below). Lachrymation was induced by placing sodium chloride (salt) crystals on the eyes, and lachrymal fluid was collected using micropipettes [43]. Clinical severity was noted every day post-challenge for 8 days, as described previously [30]. The severity scores of clinical signs of IBV were as follows: 0 = normal, 1 = infrequent sneezing (single event during observation of 1 min/vaccine group), 2 = frequent sneezing (more than one event during observation of 1 min/vaccine group), 1 = mild rales, 2 = severe rales, 2 = presence of nasal exudate. The severity scores of IBV clinical signs were recorded once a day for each chicken for 8 days post-challenge. Lachrymal fluid and tracheal swabs harvested at 6 dpc were analyzed for viral RNA using IBV N gene-specific qRT-PCR. A similar experimental design was used to test the efficacy of the pmQAC/MVA-N vaccine candidate in a follow-up trial. Before IN inoculation, 10 µg MPLA/bird (PHAD^®^, Avanti^®^ Polar Lipids, Alabaster, AL, USA) was added to the QAC-pCAG-N formulation and followed by a booster MVA-N (10^8^ pfu/bird) dose at day 14 via the intranasal (IN) route. Birds were challenged with a dose of 10^6.5^ EID_50_/bird of a virulent IBV Arkansas DPI strain via direct intranasal instillations at day 21 of age. Vaccine efficacy indicators, including viral shedding and clinical severity scoring, as detailed for the previous primary trial, were evaluated.

### 2.5. IBV Specific ELISA

To improve ELISA sensitivity, the pCAG constructs were used to produce purified Arkansas DPI S1 and N proteins as described previously [30]. Sera and lachrymal fluid from different time-points were screened for humoral response against the IBV Arkansas serotype. In order to measure IgY and IgA antibody levels in the plasma and lachrymal fluid of chickens, respectively, an IBV-specific enzyme-linked immunosorbent assay (ELISA) was developed as described previously, with modifications [44]. Briefly, ELISA plates were coated with inactivated IBV Arkansas (100 ng/well, IgY) or IBV Arkansas DPI S1 and N6xHis protein (50 ng total/well, IgA) diluted in carbonate/bicarbonate buffer with pH 9.6 and incubated overnight at 4C, followed by blocking with 5% skim milk to reduce background. A 50 µL sample of diluted serum (1/200) or lachrymal fluid (1/50) harvested at different time-points from immunized chickens was added to the wells and incubated at 37 °C for 1 h. Post-washing (PBS-TritonX 100, 0.1%) with either HRP-conjugated anti-chicken IgY (NBP1-74778, NOVUS Bio, Centennial, CO, USA) or anti-chicken IgA (NB7284, NOVUS Bio, Centennial, CO, USA) at dilutions of 1/1000 was added to the wells and incubated at 37 °C for 1 h. After washing, 50 µL of TMB substrate solution was added and incubated for 20 min or until color developed. The reaction was stopped by the addition of 1M sulfuric acid, and plates were read at 450 nm. To generate standard curves, sera and lachrymal fluid from severely IBV-infected chickens from previous experiments was used. O.D._450_ values were assigned proportional arbitrary values and used for analysis.

### 2.6. Flow Cytometric Assessment of IBV-Specific Proliferation

In a separate follow-up study, 16 chicks were divided equally into 4 groups (N = 4 each) and used for the flow cytometric assessment. The first 2 groups were inoculated with PBS (negative control) or commercial Arkansas MLV (Mildvac-Ark^®^, Merck Animal Health, Madison, NJ, USA, positive control) via direct intranasal instillations (dose according to manufacturer’s instructions). The other groups were either vaccinated with MVA-N (10^8^ pfu/bird) at day 1, followed by a booster dose at day 14 via the intranasal (IN) route, or pQAC-N (100 µg/bird) at day 1, followed by a booster MVA-N (10^8^ pfu/bird) dose at day 14 via the intranasal (IN) route. All chicks were euthanized at 20 dpv, and single cell suspensions from lungs were prepared using standard techniques and used for T-cell proliferation assay. Briefly, lungs were excised and placed in a gentleMACS dissociator M tube (Miltenyi 130-093-236) with 5 mL collagenase B (2 mg/mL, Roche, Basel, Switzerland). Lung tissue was processed using the gentleMACS dissociator, followed by incubation for 30 min at 37 °C with gentle shaking. Single-cell suspensions were prepared by gently squeezing through a 70 mm cell strainer (Falcon) after lysing RBCs using 1X BD Biosciences BD Pharm Lyse™. A total of 10^7^ cells/mL were stained with CellTrace™ Violet Cell Proliferation dye (Thermo Scientific C34557) according to the manufacturer’s instructions, and 100 µL of cells was plated/well in RPMI 1640 with 10% chicken immune serum (collected from an IBV seropositive chicken). After overnight incubation at 41 °C, 5% CO_2_, cells were stimulated with 130 ng of IBV N6xHis protein complexed with chitosan matching the challenge strain per well in 100 µL of RPMI 1640 with 10% chicken immune serum (collected from an IBV seropositive chicken). Four days post-stimulation, cells were stained for surface markers CD4- AF647 (clone CT-4) and CD8α- FITC (clone 3-298) together as well as TCRγδ-FITC (clone TCR-1) independently for flow cytometry analysis. All antibodies were purchased from SouthernBiotech (Birmingham, AL, USA). Signals were acquired on an BD LSR Fortessa flow cytometer. Data were analyzed with FlowJo software (BD Biosciences). The strategy for gating on proliferating CD4+ and CD8a+ T-cells was debris exclusion on the Forward Scatter (FSC)—Side Scatter (SSC) dot plot, followed by exclusion of dead cells by fixable viability dye eFluor 780 (Invitrogen™, #65-0865-14) staining. Out of the live cells, total proliferated cells were gated positive using a histogram plot with ef450 on the *x*-axis (for CellTrace™ Violet). Finally, CD4 cells were gated positive at the AF647 axis and CD8a cells were gated positive at the FITC axis using a FITC-AF647 dot plot. A similar approach was used for identifying proliferating TCRγδ+ T-cells. The output, stimulation index (SI), is the ratio of % proliferating cells post-stimulation to the % proliferating cells in unstimulated conditions. The chicks from different groups used herein were part of another, larger study, and the data for the control groups (PBS and MLV) have already been published [30].

### 2.7. Viral Load Measurement

RNA was extracted from lachrymal fluid (10 µL) or tracheal swabs (100 µL from total 1 mL) collected from chickens using the Zymo Direct-Zol™ RNA mini prep kit (Zymo Research, Irvine, CA, USA) according to the manufacturer’s instructions. RT-qPCR was conducted in two steps: cDNA synthesis (Invitrogen™ SuperScript™ III First-Strand Synthesis System) and qPCR reactions. cDNA synthesis was performed with 0.5 µL (50 ng/µL) random hexamers, 0.5 µL of 10 mM dNTPs, and 4 µL RNA. It was then heated at 65 °C for 5 min and chilled on ice, followed by the addition of 1 µL of 10X RT buffer, 1 µL of 0.1 M DTT, 1 µL of 25 mM MgCl_2_, 0.5 µL of RNaseOUT, and 0.5 µL of SuperScript III enzyme, for a final volume of 10 µL. The reaction conditions include 25 °C for 5 min, 50 °C for 60 min, and 70 °C for 15 min. SYBR green RT-qPCR was performed using an IBV N gene-specific primer pair, forward primer: 5′ ATGCTCAACCTAGTCCCTAGCA 3′ and reverse primer: 5′ TCAAACTGCGGATCATCACGT 3′, amplifying 128 nt of N gene of IBV Arkansas DPI. PCRs were performed using a StepOnePlus™ Real-Time PCR System (Applied Biosystems, Foster City, CA, USA) under the following conditions: one cycle at 95 °C for 2 min, followed by 40 cycles at 95 °C for 3 s and 60 °C for 30 s. Each 20 µL reaction was carried out using 1 µL of diluted cDNA (1/10), 10 µL of GoTaq^®^ qPCR mastermix (Promega, Madison, WI, USA), 2 µL of primer mixture (forward and reverse primers; 0.5 µM each) and 7 µL of nuclease free water. A serial 10-fold dilution of pCAG-IBV Ark N6xHis plasmid was used to establish the standard curve. Temperature melt curve analysis was used to confirm the specificity of the product.

### 2.8. Statistical Analysis

Statistical analyses were performed using GraphPad software (La Jolla, CA, USA). The D’Agostino–Pearson test was used to confirm normal distribution of ELISA, viral load, and clinical severity data. The Shapiro–Wilk test was used to confirm normal distribution of T-cell data. Cellular immune assays, clinical severity scoring, and viral loads were compared using an ordinary one-way ANOVA with Tukey’s multiple comparisons test, where *, *p* < 0.05; **, *p* < 0.01; ***, *p* < 0.001; ****, *p* < 0.0001 were considered significantly different among groups. Antibody titers were compared using a two-way ANOVA test, where *, *p* < 0.05; **, *p* < 0.01; ***, *p* < 0.001; ****, *p* < 0.0001 were considered significantly different among groups.

## 3. Results

### 3.1. Design and Construction of MVA-IBV Constructs

**Figure 1:**
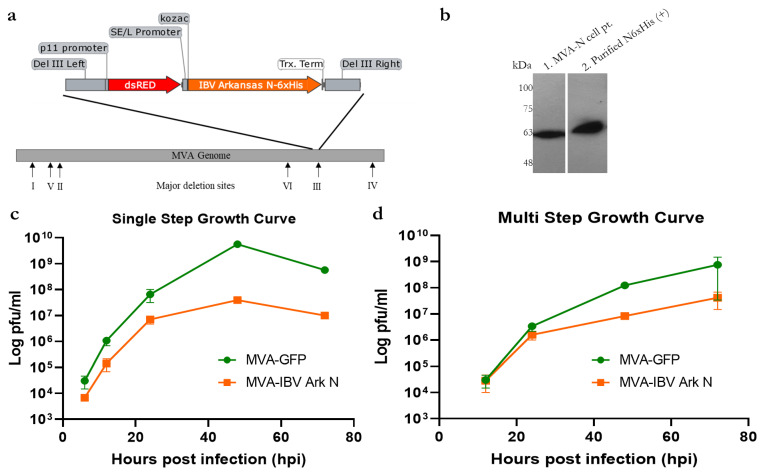
Design and characterization of MVA-IBV vaccine constructs. (**a**) MVA vaccine construct expressing N protein with the addition of C-terminal 6X His tag. Gene map was generated using Snapgene software. (**b**) Western blot analysis with anti 6xHis-HRP antibody for MVA-N confirming expression of N protein from MVA-N. Lanes are as follows: cell pellet (lane 1) from CEF cells infected with MVA-N and control purified N6xHis protein (lane 2). Cell pellet (lane 2) from CEF cells infected with MVA-N. (**c**) Single-step (MOI 1) and (**d**) multi-step growth curve (MOI 0.1) of parental MVA-GFP and recombinant MVA-N vaccine vectors. Growth curves were performed in duplicate. Data show means ± SEM.

The recombinant N-6XHis gene from pCAG-N, which encodes the nucleocapsid (N) protein from IBV Arkansas serotype with a C-terminal 6XHis tag [30] was cloned into the deletion III region within the MVA genome using an MVA shuttle vector, which enables homologous recombination and transgene insertion (Figure 1a). The SE/L promoter controls the expression of the recombinant N-6xHis protein in the MVA vaccine candidate (MVA-N, Figure 1a). Expression of N-6xHis antigen from the MVA-N vaccine construct was confirmed using Western blot analysis of proteins present in MVA-N-infected CEF cells (Figure 1b). To characterize and understand whether the expression of IBV N-6xHis protein affected MVA replication in the cell culture, we evaluated the replication kinetics of MVA-N and parental MVA-GFP in permissive CEF cells. CEF cells were either infected at a MOI of 1 (single-step) or 0.1 (multi-step), and viral titers subsequently determined on CEF cells (Figure 1c,d), although a higher MOI of 5 might have been required to infect all cells in order to generate a single-step growth curve. MVA-N initially replicated at rates similar to parental MVA-GFP, although the final titers of the MVA-N were about 100-fold lower than those of the parental virus (Figure 1c,d).

### 3.2. Heterologous Vaccine Strategy Elicits Robust Localized T-Cell Responses

**Figure 2:**
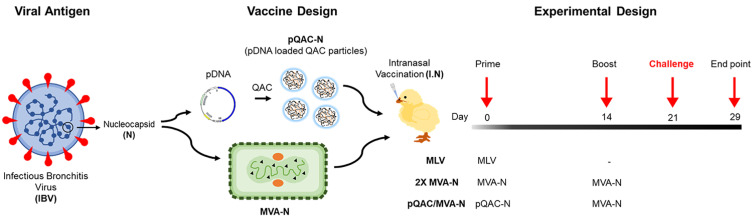
Experimental design of IBV immunization and challenge studies. Outline for vaccine construct and immunization protocol using groups of white leghorn SPF birds vaccinated with MVA-N (IN) or pQAC-CoV (IN) at day 0, followed by boost with MVA-CoV (IN) at day 14. Control groups include unvaccinated the PBS group and the group who received commercial MLV vaccination at day 0 (IN).

We have previously reported the safety and protective efficacy of QAC complexed pCAG-N DNA vaccine (pQAC-N) in chickens against an IBV Arkansas challenge, although no humoral responses were observed [30]. We hypothesized that a heterologous mucosal strategy of priming with pQAC-N followed by boosting with MVA-N would offer a similar or better level of protection than that observed with two-dose intranasal (IN) pQAC-N vaccination with complementing humoral responses. For this purpose, groups of white leghorn specific pathogen-free (SPF) chickens were either unvaccinated (PBS, negative control) or vaccinated with the commercial Arkansas modified live virus (MLV, positive control) vaccine (Figure 2). One group of SPF chickens was immunized with MVA-N at day 1, followed by a boost at day 14 post-initial immunization (homologous vaccine platform group, 2X MVA-N). Another experimental group was immunized with pQAC-N at day 1, followed by an MVA-N boost at day 14 post-initial immunization (heterologous vaccine platform group, pQAC/MVA-N). All immunizations were administered via the IN route, as indicated above (Figure 2).

**Figure 3:**
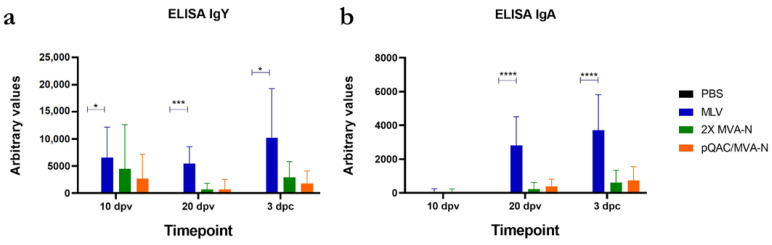
Humoral immune responses in vaccinated chicks. IBV-specific (**a**) IgY in serum and (**b**) IgA in lachrymal fluid, significance (*, *p* < 0.05; ***, *p* < 0.001; ****, *p* < 0.0001) was determined by two-way ANOVA. Data show means ± SEM.

We examined the ability of our experimental vaccines to elicit local and systemic IBV- specific immune responses following IN immunization. Lachrymal fluid samples and serum harvested at different time points, 10 and 20 days post-vaccination (dpv, pre-challenge) and three days post-challenge (dpc), were examined for IBV-specific IgA (lachrymal fluid, local) and IgY (serum, systemic) using ELISA. IBV-specific IgA and IgY were significantly higher in the MLV group when compared to the unvaccinated PBS group (Figure 3a,b). Although they were detectable at multiple time points, both IgA and IgY levels were not significantly higher in birds vaccinated with either the homologous or heterologous vaccine strategy than in unvaccinated birds (Figure 3a,b).

**Figure 4:**
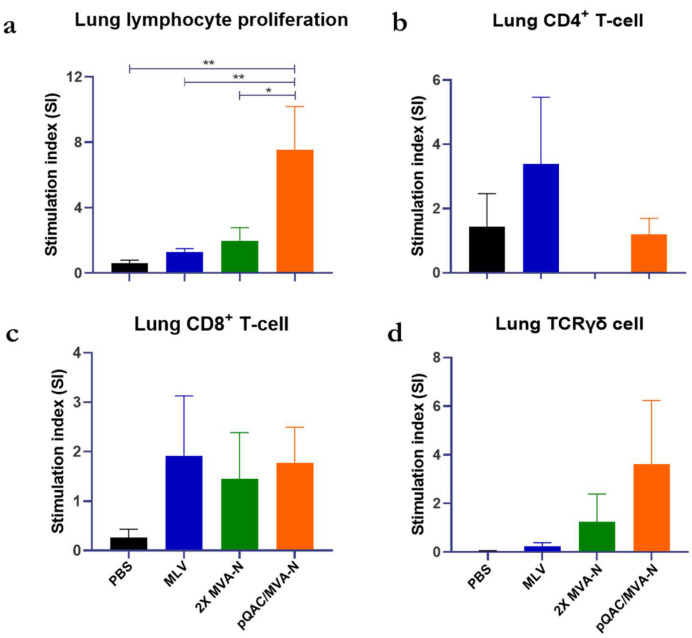
Localized T-cell immune responses in vaccinated chicks. Lung cell proliferative capacity measured by CellTrace Violet dye dilution in unvaccinated, MLV, 2X MVA-N, and pQAC/MVA-N vaccinated chickens. Proliferation was measured in (**a**) total lung cells, (**b**) CD4+, (**c**) CD8+, and (**d**) TCRγδ+ lung T-cells after 4 days in culture post-stimulation with IBV N6xHis protein, matching the IBV Arkansas DPI challenge strain complexed with chitosan. Non-significance (ns) or significance (*, *p* < 0.05; **, *p* < 0.01) was determined by one-way ANOVA with multiple comparisons. Data show the means ± SEM.

We next evaluated the ability of the experimental vaccines to elicit local (lung) IBV N-specific cellular immune responses. An antigen-specific T-cell proliferation assay based on CellTrace™ Violet Cell dye staining of lung cells to trace proliferating T-cells was used as described previously [30]. Using this flow cytometry-assisted T-cell assay, different T-cell subsets responding to recall antigen stimulation were identified. Twenty days post-initial immunization, in an independent study, lungs from chickens from each group were harvested, and cell proliferation in response to IBV N-antigen stimulation was measured. The stimulation index (SI), which is the fold increase in stimulated to unstimulated cells, was calculated. Total lung cells from pQAC/MVA-N-vaccinated birds had significantly higher proliferation in response to N antigen stimulation than the control and 2X MVA-N groups (Figure 4a). An increase in the proliferating TCRγδ+ and CD8+ T-cells in response to N antigen stimulation was observed in pQAC/MVA-N-vaccinated birds in comparison to control birds (Figure 4c,d), while CD4+ T-cell proliferation was higher in MLV-vaccinated birds (Figure 4b), albeit non-significant. These results highlight the ability of the heterologous pQAC/MVA-N vaccine strategy to elicit robust IBV-specific immune responses.

### 3.3. The Heterologous Vaccine Strategy Is More Effective Than the Homologous Vaccine Strategy

**Figure 5:**
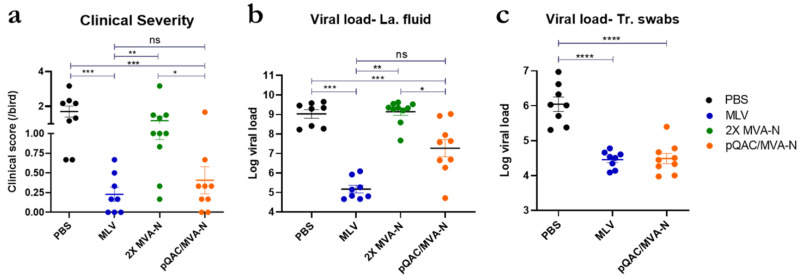
Increased protection with heterologous vaccine strategy against IBV. (**a**) Clinical sign severity, represented as average score/bird over 8 days post-challenge in each group. (**b**) IBV log viral load (genome copy no)/10 µL lachrymal fluid at 6 days post-challenge. (**c**) IBV log viral load (genome copy no) in tracheal swab at 6 days post challenge. Non-significance (ns) or significance (*, *p* < 0.05; **, *p* < 0.01, ***, *p* < 0.001; ****, *p* < 0.0001) was determined by one-way ANOVA with multiple comparisons. Data show the means ± SEM.

Twenty-one days post-initial vaccination (dpv) and seven days post-final boost, immunized birds were challenged with a virulent strain of IBV Arkansas serotype (Arkansas DPI genotype) via the intranasal route to evaluate vaccine efficacy. The clinical severity of the birds was evaluated up to eight days post-challenge (dpc), and lachrymal fluid and tracheal swabs were harvested at six dpc to evaluate the viral burden. Immunization with homologous 2X MVA-N did not confer any protection against the challenge; no reduction in clinical severity was observed (Figure 5a). In contrast, immunization with heterologous pQAC/MVA-N and MLV resulted in a significant reduction in clinical severity when compared to birds in the unvaccinated PBS group (Figure 5a). Viral RNA in lachrymal fluid and tracheal swabs was evaluated using qRT-PCR. Only the best-performing experimental vaccine group, as determined by viral shedding in lachrymal fluid along with the control groups, was taken for quantifying viral shedding in the tracheal swabs. A significant reduction in viral load was observed both in the lachrymal fluid and swabs of pQAC/MVA-N-vaccinated birds in comparison to the unvaccinated and 2X MVA-N-vaccinated birds (Figure 5b). More importantly, the reduction in viral load in tracheal swabs was comparable to levels seen in commercial MLV-vaccinated birds (Figure 5c). In contrast, no reduction in viral load was observed in 2X MVA-N-vaccinated birds, which correlated well with clinical severity scoring (Figure 5a,b). Vaccination with the heterologous pQAC/MVA-N conferred significantly higher protection against the IBV challenge than the homologous 2X MVA-N (Figure 5b). This protection might be attributed to the induction of robust cell-mediated memory responses mediated by pQAC/MVA-N in the lungs (Figure 4).

### 3.4. Impact of MPLA Addition on IBV Vaccine Protection

**Figure 6:**
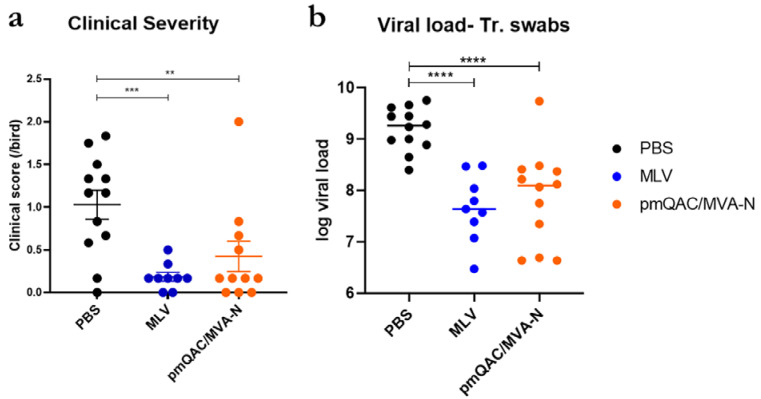
Protective efficacy of the MPLA-QAC triple adjuvant system. (**a**) Clinical sign severity, represented as average score/bird over 8 days post-challenge in each group. (**b**) IBV log viral load (genome copy no) in tracheal swab at 6 days post-challenge. Significance (**, *p* < 0.01; ***, *p* < 0.001; ****, *p* < 0.0001) was determined by one-way ANOVA with multiple comparisons. Data show the means ±SEM.

MPLA is a potent mucosal adjuvant and TLR 4 ligand that stimulates the expression of inflammatory-related genes; this is important for viral control in poultry. We hypothesized that inclusion of MPLA in addition to Quil-A and chitosan would further improve the protection observed with pQAC/MVA-N vaccination. To investigate this, we immunized SPF birds with a triple adjuvant system (MPLA +QAC) loaded with pCAG-N plasmid at day 1, followed by MVA-N immunization (pmQAC/MVA-N) at day 14, similarly to the pQAC/MVA-N group in the previous trial. A reduction in clinical severity and viral burden in tracheal swabs was observed to be comparable to the MLV group (Figure 6a,b). The protective efficacy of pmQAC/MVA-N was very similar to and not significantly different from pQAC/MVA-N (Figure 6a,b). Our results indicate that addition of MPLA does not improve vaccine performance, although direct comparisons with pQAC/MVA-N are required to conclusively evaluate the effects of addition of MPLA. Overall, these results highlight the ability of the heterologous vaccine strategy to elicit potent IBV-specific T-cell responses and protect vaccinated birds against a virulent IBV challenge.

## 4. Discussion

Many experimental viral vectored vaccines, primarily based on Newcastle Disease Virus (NDV), have been developed against IBV [31,45,46]. Recombinant NDV encoding IBV spikes protect against homologous challenge and result in a reduction in clinical severity and viral shedding [31,46]. Recombinant MVA-based vaccines have been developed for use in chickens against infectious bursal disease virus (IBDV) and influenza [47,48,49]. The heterologous vaccine strategy, involving a DNA prime followed by a viral vector booster dose, has been evaluated against multiple human and animal viruses with modest success [35,36,37]. Intranasally administered vaccines are highly favorable for mass vaccination in the field. Unfortunately, mucosal surfaces are vaccine-hostile, leading to poor immunogen uptake and bioavailability, rapid degradation, and weak immune responses [27]. In a previous study, we demonstrated the ability of a nano-adjuvant system, QAC, to facilitate the intranasal delivery of DNA immunogens, leading to a protection against IBV challenge [30]. In this study, we evaluated the efficacy of an intranasally delivered heterologous QAC-complexed DNA prime-MVA boost vaccine strategy. To our knowledge, the use of heterologous and MVA-based vaccine strategies against coronaviruses have not been extensively studied, especially in the context of IBV infection in chickens.

DNA viral vectors such as MVA can accommodate and stably express multiple foreign immunogens, making them ideal candidates for vaccine use. In our hands, although the recombinant MVA-N initially had similar replication rates in cell-culture when compared to the parental MVA-GFP, the titers were 100-fold lower, although the differences were not statistically significant. This could mean that constitutive expression of the IBV N-6xHis protein potentially weakened the MVA vector replication in permissive CEF cells. The safety and efficacy of MVA-based vaccines in chicken hosts have been well documented [50,51,52]. Experimental MVA-hemagglutinin-based influenza vaccines protect chickens against both lethal high- and low-pathogenicity avian influenza [50,51]. Furthermore, the safety and replication of MVA in chicken embryos have been extensively characterized, with no embryonic death observed even after in ovo inoculation [52]. We have previously shown that QAC-based DNA vaccines are well-tolerated by chicken hosts when administered via the IN and in ovo routes. Similarly, we observed that chickens that were intranasally administered MVA-N and pQAC/MVA-N did not show any signs of respiratory distress, inappetence, or depression pre-challenge.

Very few studies have investigated the efficacy of MVA-based vaccines in poultry. Ocular administration of an MVA-based flu vaccine has been shown to protect birds against an avian influenza challenge [48]. MVA-based SARS-CoV-2 S vaccines are protective and induce robust neutralizing antibodies and T-cell responses in vaccinated mice and macaques [53,54]. Mixing and matching viral vector and nucleic acid SARS-CoV-2- vaccines also boost the immunogenicity of otherwise homologous vaccine platforms [55,56]. In our hands, the heterologous DNA prime followed by MVA boost was more immunogenic and protective than the prime–boost homologous MVA vaccination. Reductions in clinical severity and viral burden, both in lachrymal fluid and tracheal swabs, were observed at levels comparable with MLV vaccination. The protection is most likely due to the induction of local lymphocyte responses by the pQAC-N priming, followed by the expansion of T-cells facilitated by the MVA-N boost. We observed a similar phenomenon with our QAC-based COVID-19 vaccines in mice, where the heterologous DNA/MVA vaccine strategy was more immunogenic than the homologous vaccine strategy [57].

In a previous study, we showed that two doses of the pQAC-N vaccine protected vaccinated SPF and commercial birds against an IBV challenge at a rate comparable to the protection observed with MLV [30]. A robust T-cell immune response without a complementing humoral response was induced post-vaccination with 2X pQAC-N. We hypothesized that boosting with the MVA viral vector instead of a DNA vaccine would further expand CD4+ T-cells, leading to an induction of complementing humoral responses. We observed that immunization with MVA-N, in both the homologous and heterologous platforms, did not lead to significant induction of either IgY or IgA, as assayed using IBV specific binding ELISA. Instead, low-level IBV-specific IgA and IgY were observed in the experimental vaccine groups at 3 dpc, indicating the presence of an anamnestic response with pQAC-N-based vaccines. In contrast, significant induction of humoral responses was observed with the commercial MLV vaccine. Irrespective of the vaccine platform used, either the homologous MVA and heterologous DNA/MVA used in this study or the homologous DNA used in the previous study, significant induction of N-specific humoral responses were not observed [30]. The absence of humoral responses could be a consequence of using the N immunogen exclusively and not the vaccine platform itself. The N protein here will be intracellularly expressed in cells that take up the vaccine, and not secreted. Moreover, it is unlikely that antibodies generated against N will be neutralizing, given the intra-virion nature of the protein. Specifically for IBV, immunization with N protein does not elicit IBV neutralizing antibodies [58].

Previously, sequential immunization approach of DNA prime–viral vector boost has led to the initial induction of cell-mediated immune (CMI) responses, followed by expansion of induced CD8+ T-cells and Th1 T-cells in response to the MVA boost [59]. To evaluate vaccine-induced T-cell responses, we used a flow cytometry-assisted lymphocyte proliferation assay to identify and quantify subsets of lung T-cells responding to the IBV N protein. We have previously shown that the potency of unadjuvanted plasmid DNA vaccine was enhanced by QAC nanoparticle formulation, leading to the induction of robust CD8+ and TCRγδ+ T-cells, as is potentially a hallmark of the QAC adjuvant system [30]. Similarly, lung cells harvested from pQAC/MVA-N-immunized chickens responded well to IBV-N antigen recall stimulation. Significant stimulation of total lung cells was observed with the heterologous vaccination strategy, but not with the homologous MVA-N vaccination, clearly highlighting the ability of the heterologous platform to expand cell-mediated immune responses. Furthermore, higher stimulation of TCRγδ+ and CD8+ T-cells was observed in pQAC/MVA-N immunized chickens, albeit statistically non-significant. In contrast, stimulation of CD4+ T-cells was observed only with MLV lung cells. Although no significance was observed in T-cell specific responses, statistically higher proliferation was observed with total lung cells. This could mean that there are other lymphocytes (non TCRγδ+, CD8+ or CD4+ T-cells) in the lungs responding to the IBV antigen that were not specifically evaluated in this study. We believe that an MVA boost after a DNA prime further expanded the lung lymphocytes elicited by the initial DNA vaccination, leading to protection. These results are in accordance with our previous study, in which a similar heterologous DNA/MVA vaccine platform elicited better local type-1 and type-17 T-cell responses in mice that were not observed with the homologous vaccine strategy delivered via the IN route [57]. For the stimulation, we used N protein, complexed with chitosan, to improve the sensitivity of the cell-proliferation assay. T-cell activation in response to chitosan is limited without the antigen, and chitosan alone is insufficient to trigger a robust T-cell response [60]. That being said, further experiments are needed to clearly show that the pQAC/MVA-N-induced T-cell responses observed here were specific to the N protein and not chitosan.

To further improve upon the efficacy of the pQAC/MVA-N vaccine, we added MPLA to our QAC vaccine formulation. MPLA is a synthetic, low-toxic form of LPS that can engage with TLR4 (toll-like receptor), leading to an enhanced Th1 response [61]. MPLA is the only licensed TLR agonist approved for human use and is currently used as part of the AS04 adjuvant in hepatitis B and human papillomavirus vaccines [62,63]. Engagement of TLRs by agonists such as lipopolysaccharides (LPS), Poly I:C, and CpG dinucleotides leads to a cascade of intracellular signaling and, thus, to the induction of factors and cytokines which enhance immunity [64]. We investigated the addition of MPLA to QAC to enhance the protection observed with pQAC/MVA-N. The new tri-adjuvant system-based heterologous vaccine, dubbed pmQAC/MVA-N, did not significantly improve the protection observed with pQAC/MVA-N when administered intranasally, although we did not directly compare the efficacy of pQAC/MVA-N and pmQAC/MVA-N in a single experiment.

In general, CD8+ T-cells are important for early protection against IBV infection, but CD4+ T-cells and systemic humoral responses are needed for sterilizing long-term immunity [65]. We did not observe IBV-specific antibody responses with the heterologous vaccine. The use of additional adjuvants and a secreted IBV S protein as an additional immunogen to the pQAC/MVA-N formulation could help in generating a complementing humoral immune response [66]. Two-dose vaccine regimens such as the heterologous vaccine strategy described herein might also have poor field applicability. Single-dose vaccines administered at day 1 are preferred for poultry, considering the need for early protection against IBV and the short lifespan of broilers in the poultry industry. Many experimental MVA-based vaccines for use in humans are currently undergoing clinical trials. Therefore, the use of MVA in poultry might confer pre-existing immunity against the viral vector to people coming in contact with vaccinated birds, thus limiting the efficacy of subsequent human MVA-based vaccines.

The results presented herein highlight the utility of a nano-adjuvant-complexed DNA prime/viral vector boost vaccine strategy against IBV in chickens, which reduces clinical severity and viral load in the trachea and lachrymal fluid. The heterologous vaccine strategy outperformed the homologous MVA/MVA immunization and resulted in the induction of local-IBV-specific T-cells in the lungs. Moreover, the protection observed with the heterologous vaccine strategy was comparable with the commercial MLV vaccine’s efficacy. Importantly, the utility of this heterologous vaccine platform can be extended for use against other respiratory coronaviruses that necessitate robust local immune responses for protection. As highlighted with the ongoing COVID-19 pandemic, mix-and-match heterologous vaccines can not only improve immunogenicity, but also help in mitigating global vaccine supply chain shortages.

## Data Availability

All data are available upon request. Please contact: adel.talaat@wisc.edu.
